# One-Day Prostate Cancer Diagnosis: Biparametric Magnetic Resonance Imaging and Digital Pathology by Fluorescence Confocal Microscopy

**DOI:** 10.3390/diagnostics12020277

**Published:** 2022-01-21

**Authors:** Ugo Giovanni Falagario, Oscar Selvaggio, Francesca Sanguedolce, Paola Milillo, Maria Chiara Sighinolfi, Salvatore Mariano Bruno, Marco Recchia, Carlo Bettocchi, Gian Maria Busetto, Luca Macarini, Bernardo Rocco, Luigi Cormio, Giuseppe Carrieri

**Affiliations:** 1 Department of Urology and Organ Transplantation, University of Foggia, 71122 Foggia, Italy; oscarsel@libero.it (O.S.); mariano.bruno91@gmail.com (S.M.B.); marco.recchia291292@gmail.com (M.R.); carlo.bettocchi@unifg.it (C.B.); gianmaria.busetto@unifg.it (G.M.B.); luigi.cormio@unifg.it (L.C.); giuseppe.carrieri@unifg.it (G.C.); 2 Department of Pathology, University of Foggia, 71122 Foggia, Italy; francesca.sanguedolce@unifg.it; 3 Department of Radiology, University of Foggia, 71122 Foggia, Italy; paola.milillo@yahoo.com (P.M.); luca.macarini@unifg.it (L.M.); 4 Department of Urology, ASST Santi Paolo e Carlo Dipartimento di Scienze della Salute, Università degli Studi di Milano, 20142 Milano, Italy; sighinolfic@yahoo.com (M.C.S.); bernardo.rocco@gmail.com (B.R.); 5 Department of Urology, Bonomo Teaching Hospital, 76123 Andria, Italy

**Keywords:** biparametric magnetic resonance imaging, digital pathology, fluorescence confocal microscopy, prostate cancer

## Abstract

In this prospective observational study, we tested the feasibility and efficacy of a novel one-day PCa diagnosis path based on biparametric magnetic resonance (bpMRI) and digital pathology by fluorescence confocal microscopy (FCM). Patients aged 55–70 years scheduled for PBx due to increased PSA levels (3–10 ng/mL) and/or abnormal digitorectal examination were enrolled. All patients underwent bpMRI and PBx with immediate FCM evaluation of biopsy cores. Patients were asked to fill out a dedicated Patient Satisfaction Questionnaire. Patients’ satisfaction rates and concordance between digital pathology and standard HE evaluation were the outcomes of interest. Twelve patients completed our one-day PCa diagnosis path. BpMRI showed suspicious lesions in 7 patients. Digital pathology by FCM identified PCa in 5 (41.7%) of the 12 patients. Standard pathology confirmed the diagnosis made through digital pathology in all the cases. At a per patient level, high concordance between the methods was achieved in Gleason Grading (4 out of 5 patients). The level of agreement in the number of positive cores was lower but did not affect the choice of treatment in any of the 5 PCa cases. At a per core level, the agreement was very high for the diagnosis of anyPCa (96.2%) and csPCa (97.3%), with a k coefficient of 0.90 and 0.92, respectively (near perfect agreement). In conclusion, one-day PCa diagnosis by FCM represents a feasible, reliable, and fast diagnostic method that provides significant advantages in optimizing time and resources, leading to patients having a higher quality standard of care perception.

## 1. Introduction

Worldwide, prostate cancer (PCa) screening and diagnosis path is a major health concern [1]. In fact, while screening by prostate-specific antigen (PSA) assessment has been shown to allow for earlier detection of clinically significant disease, the risk of overdiagnosis and overtreatment limits its implementation at a population level [1,2,3]. A single elevated PSA should not prompt immediate prostate biopsy (PBx) [4]; conversely, the decision to biopsy a man with an elevated PSA should be based on numerous factors, including repeat blood draw for confirmatory testing of the PSA level, digitorectal examination (DRE), and work-up for benign disease [4]. In order to reduce the number of unnecessary PBxs, the European Urological Association (EUA) guidelines recommend that, even after these steps, asymptomatic men with a normal DRE and a PSA level between 2 and 10 ng/mL should be offered further risk assessment prior to performing a PBx [5]. Available tools include novel biomarkers [6,7,8], risk calculators [9], and multi-parametric prostate MRI (mpMRI) [10]. The latter has the advantage of not only reducing the number of PBxs performed but also guiding prostate sampling to suspicious areas [11,12].

In this scenario, a patient presenting with a first elevated PSA usually waits up to 8 weeks from the decision to perform PBx to the final histological diagnosis [13]. Performing pre-biopsy mpMRI, as recommended by EAU guidelines, may result in an additional delay. While questions remain unanswered regarding the impact of the waiting time on the prognosis of patients treated for PCa, it certainly impacts on patients’ quality of life and healthcare quality perception, which are recognized as key indicators of healthcare quality. According to WHO, “health care must be safe, effective, timely, efficient, equitable and people-centred”. As such, reducing waiting times and improving patients ‘experience (especially for cancer diagnosis and treatment) has become a priority and it is imperative to find new diagnostic strategies that hesitate in fast and accurate diagnosis.

Recent advances in imaging protocols [14,15,16] and a novel digital pathology method using fluorescence confocal microscopy (FCM) that does not require paraffin embedding and sectioning [17] hold promise for reducing waiting times from suspicion to diagnosis and treatment of PCa. In order to deliver the best quality of care available in a timely manner, we developed a pathway for one-day PCa diagnosis based on biparametric non-contrast-enhanced prostate MRI (bpMRI) and digital biopsy using FCM. Herein, we report our preliminary data on the methods, results, and patients’ satisfaction achieved using this novel diagnostic pathway.

## 2. Materials and Methods

This study was a prospective single-center pilot study evaluating the feasibility and efficacy of a one-day PCa diagnosis pathway. This study was conducted in December 2020 and enrolled patients aged 55–70 years scheduled for PBx at our institution due to increased PSA levels (up to 10 ng/mL) and/or abnormal DRE. Men receiving 5 alfa-reductase inhibitors (5-ARIs) or who had previously undergone prostate MRI or PBx or an invasive treatment for benign prostate hyperplasia (BPH) were excluded. Supplementary Figure S1 graphically summarizes the proposed one-day PCa diagnosis pathway. Specifically, eligible patients were scheduled to undergo uroflowmetry and ultrasound evaluation of post void residual urinary volume (PVR), as per our protocol [18,19,20], biparametric prostate MRI, PBx, digital pathology by FCM, and pre-discharge clinical assessment. The study protocol was approved by the University of Foggia Ethics Committee and written informed consent to take part was given by all participants.

### 2.1. Biparametric MRI

All patients underwent prostate MRI with a 1.5 T MR scanner (Achieva, Philips Healthcare, Best, The Netherlands) without the use of contrast agents and without endorectal coil, meaning only surface array coils were used. The bpMRI protocol consisted of Turbo-Spin-Echo (TSE) T2-weighted imaging in the axial, coronal, and sagittal planes (repetition time (TR) 5300, echo time (TE) 150 ms, slice thickness 3 mm, field of view (FOV) 180 × 180, number of signal averaged (NSA) 8); B. TSE T1-weighted imaging in the axial plane (TR/TE 400–650/12 ms, thickness 3 mm, FOV 180 × 180, NSA 3); C. diffusion-weighted imaging sequence (DWI) in the axial plane (TR/TE 3481/92 ms, slice thickness 3 mm, FOV 180 × 220, NSA 4, b-values 0–500–1000–1500 s/mm^2^). All exams were evaluated by a dedicated uro-radiologist with 7 years of experience in prostate MRI. MRI volume was used to compute PSA density.

### 2.2. Prostate Biopsy

Following local anesthesia [21], all patients underwent a 14-core transrectal systematic biopsy according to our institutional protocol [22]. In patients with a positive bpMRI, two to four additional cores from the suspicious lesions were obtained using an Electromagnetically Tracked MRI/US Fusion system (Navigo, UC-Care).

### 2.3. Pathology

The biopsy cores were evaluated by FCM immediately after having completed prostate sampling. The microscope (VivaScope 2500M-G4) was located in a dedicated room next to the room where biopsy was performed. Preparation of the samples and FCM digital image acquisition were carried out by two urologists following the manufacturer’s validated protocols (Supplementary Materials). In order to reduce the preparation and acquisition time, each glass slide was loaded with three biopsy cores. Each slide was then scanned for a maximum time of 2 min and then stored on a dedicated hard disk, according to a patient identification number, biopsy date, and image code. The FCM device combines two different lasers that enable tissue examination according to reflectance (785 nm) and fluorescence (488 nm) modalities. Images were rendered as pseudo-hematoxylin-eosin (HE) images, relying on the combination of two images acquired at each wavelength. The fresh samples were then formalin-fixed and sent to the pathology department for standard HE evaluation. A single dedicated uropathologist blind to the clinical data evaluated the pseudo-HE digital images on the day of PBx, assessing in each core the presence of any PCa (Gleason Group (GG) ≥1) and clinically significant PCa (csPCa, GG ≥ 2). The same pathologist reported standard HE-processed biopsies according to the ISUP recommendation [23]. The definitive report was ready two weeks after the procedure.

### 2.4. Pre-Discharge Clinical Assessment

As per our protocol, all patients were kept in the outpatient clinic to assess their ability to void and the PVR after PBx. At discharge, they were informed of the digital pathology results and treatment perspectives, with the aim of further discussing them at the time of the final pathology results. Patients with digital diagnosis of high-grade (GG 4-5) PCa were scheduled to undergo staging imaging, with the aim of discussing it by the time of the final pathology results.

### 2.5. Patients’ Satisfaction Questionnaire

On the day of PBx, patients were asked to fill out, before leaving the outpatient clinic, a dedicated Patient Satisfaction Questionnaire. The survey included 7 items about the different aspects of care in patients with suspicion of PCa: 1. Time from suspicion to histological diagnosis; 2. Time from biopsy to histological diagnosis; 3. Workdays lost for PCa evaluation; 4. Reducing hospital visit during COVID-19 outbreak; 5. Prostate MRI; 6. Urological physical examination; 7. Blood tests. Each item was scored with two subscales, ranging between 1 and 5, to assess perceived importance (not important (1) to extremely important (5)) and satisfaction with medical care (unsatisfied (1) to extremely satisfied (5)). Results were collected anonymously.

### 2.6. Study Outcomes and Statistical Analysis

The primary objective of the present study was to test the feasibility and efficacy of one-day PCa diagnosis based on bpMRI and digital biopsy using FCM. Efficacy was assessed on a per-patient basis and per-core basis in terms of the agreement between diagnosis of anyPCa (using digital FCM imaging and in terms of patients’ satisfaction.

As a secondary analysis, Cohen’s kappa coefficient was used to evaluate the intra-rater reliability for each biopsy core (ratings per core = 2) for the detection of anyPCa (number of rating categories = 2: Negative vs. GG 1-2-3-4-5), CsPCa (number of rating categories = 2: Negative and GG 1 vs. GG 2-3-4-5), and individual GG (number of rating categories = 6: Negative vs. GG 1 vs. GG 2 vs. GG 3, vs. GG 4, GG 5). The Kappa statistic varies from 0 to 1, where: 0 = agreement equivalent to chance, 0.1–0.20 = slight agreement, 0.21–0.40 = fair agreement, 0.41–0.60 = moderate agreement, 0.61–0.80 = substantial agreement, 0.81–0.99 = near perfect agreement, 1 = perfect agreement. Statistical analyses were performed using Stata-SE 15 (StataCorp LP, College Station, TX, USA) using the following syntax: kappaetc.

## 3. Results

In total, 12 patients were enrolled in our one-day PCa diagnosis pathway with a total of 185 biopsy cores taken and analyzed using both diagnostic modalities.

**Figure 1:**
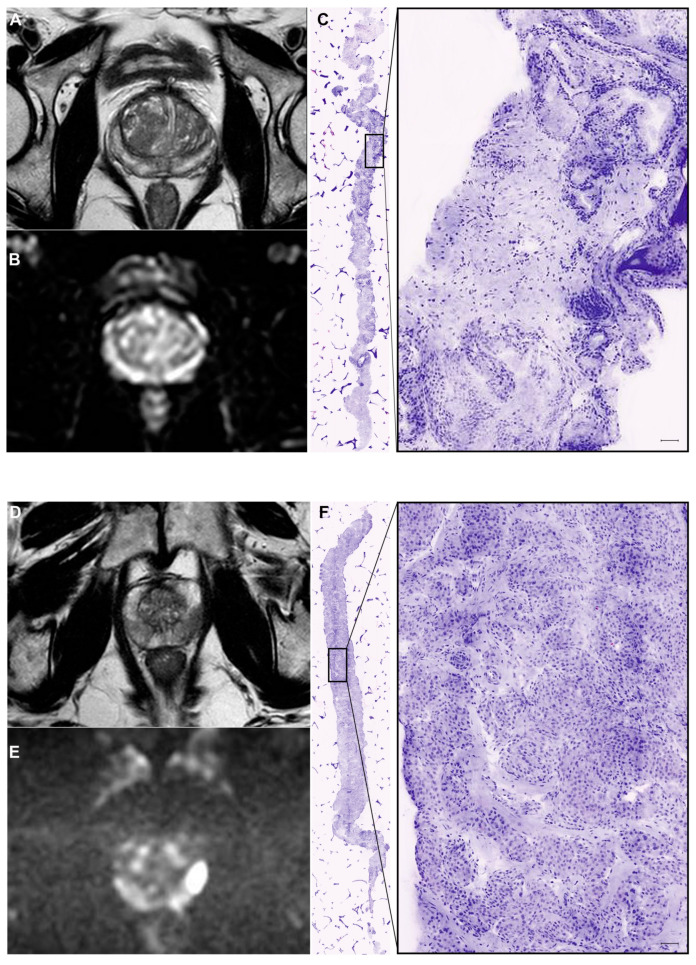
Representative MRI and digital pathology images of two patients included in the study. (**A**–**C**) Patient 10: PSA 8.77 ng/mL, Suspicious digitorectal examination (DRE), negative MRI ((**A**): T2W image; (**B**): DWI Image), negative digital biopsy (**C**). (**D**–**F**) Patient 5: PSA 5.14 ng/mL, negative DRE, suspicious MRI (PIRADS 4; (**D**): T2W image; (**E**): DWI image), positive digital biopsy (**F**).

Eleven patients received the full schedule since one refused prostate MRI due to a panic attack in the radiology department. Representative MRI and digital pathology images of two patients included in the study are presented in Figure 1. The characteristics of the enrolled patients are summarized in Table 1. The median age was 60 years, median PSA was 5.1 ng/mL, and 7 (58%) patients had a positive DRE. Three patients had severe lower urinary tract symptoms (IPSS > 19) and reduced peak flow rate at uroflowmetry (<10 mL/s).

BpMRI showed suspicious lesions in seven patients including two patients who had PI-RADS 5 lesions with highly suspicious prostatic capsule involvement.

### 3.1. Per-Patient Analysis

Digital pathology by FCM identified PCa in 5 (41.7%) of the 12 patients, with 4 of the 5 cancers being csPCa. Standard pathology confirmed PCa in all 5 cases diagnosed at digital pathology, with concordance in GG in 4 of the 5 patients and discordance in 1, whereby the GG 4 diagnosed at digital pathology was upgraded to GG 5 on standard HE pathology. Agreement in the number of positive cores was obtained in one patient, whereas there was disagreement, generally on one core, in the rest of the cohort. Such a difference in the number of positive cores did not affect the choice of treatment in any of the five patients with PCa. Active surveillance was proposed to one patient, robotic radical prostatectomy to two patients, and an 18F-CholinePET/CT was suggested and scheduled for the remaining two patients to stage their high-grade PCa.

### 3.2. Per-Core Analysis

Crosstabulation of the per-core GG assessment using digital FCM and standard of care (HE staining) is presented in Table 2. The agreement was very high for the diagnosis of anyPCa (96.2%) and csPCa (97.3%), with a k coefficient of 0.90 and 0.92, respectively (near perfect agreement). The agreement in GG assessment was lower (90.8%), with a K coefficient of 0.80 (substantial agreement). Notably, 5 negative cores according to digital FCM (2.7%) were upgraded to cancer on HE staining, with a final diagnosis of GG1 in 2 cores, GG3 in 2 cores, and GG4 in 1 core. Conversely, 2 negative cores according to HE staining were graded as GG1 at FCM evaluation.

### 3.3. Patient Satisfaction Questionnaire

**Figure 2:**
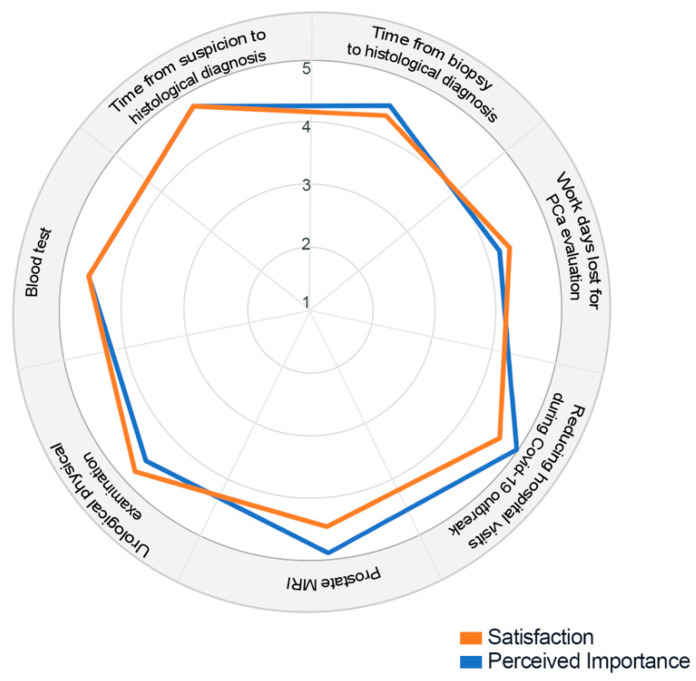
Radar chart of the survey results. The figure represents the mean rating values of satisfaction with medical care and perceived importance about different aspects of care for patients with suspicion of prostate cancer (PCa): 1. Time from suspicion to histological diagnosis; 2. Time from biopsy to histological diagnosis; 3. Workdays lost for PCa evaluation; 4. Reducing hospital visit during COVID-19 outbreak; 5. Prostate MRI; Urological physical examination; Blood tests.

The Patient Satisfaction Questionnaire results of the 12 patients included in the study are reported in Table S1 and graphically presented using a radar chart (Figure 2).

The aspects with the highest perceived importance for patients included in the study were item 4 (reducing hospital visit during the COVID-19 outbreak) and item 5 (prostate MRI). In total, 11 and 10 patients scored to reduce time from suspicion to histological diagnosis (item 1) and from biopsy to histological diagnosis (item 2) as extremely important, respectively. Patients’ satisfaction rates were high to very high for all the evaluated aspects.

## 4. Discussion

The feasibility of a “one-stop” diagnostic PCa pathway including multiparametric MRI (mpMRI) and transperineal targeted biopsy has already been shown in a pilot study and is currently under evaluation on a larger scale by the UK’s National Health Service. This pilot study indicated that such an approach reduced times to diagnosis and treatment to a median of 8 and 20 days, respectively [24].

Based on recent evidence suggesting no difference in the cancer detection rates between bpMRI and mpMRI in biopsy-naïve patients [25,26,27], and based on the results of the first trial evaluating the efficacy of a fast bpMRI-based PCa screening program (PROSTAGRAM: acquisition time 15 min) [28], we elected to use bpMRI due to its advantages of reducing acquisition times and costs. Moreover, bpMRI prevents the potential risks associated with contrast administration, is not affected by renal function, and requires limited preparation.

The real novelty of our study, however, was introducing real-time digital pathology by FCM, thus shifting the paradigm from “one-stop” to “one-day” diagnosis of PCa. The findings were clear: all Pca cases were identified by digital pathology and there was a high concordance in the GG definition between digital and standard pathology. Concordance in the number of positive cores was lower but did not impact on the treatment decision-making process. In other words, in this limited number of cases, standard pathology did not modify the treatment strategy arising from digital pathology assessment. The immediate impact of this novel pathway was high patient satisfaction as clearly pointed out by our dedicated satisfaction questionnaire. Receiving the best quality of care available in one day (i.e., imaging, blood tests, and physical examination), thus reducing hospital visits and time from suspicion to diagnosis, emerged as the aspects with the highest perceived importance.

This novel paradigm of “one-day” diagnosis of Pca has several potential advantages. First, reducing the waiting time from suspicion to diagnosis of PCa decreases anxiety while improving patients’ experiences and satisfaction with health care. Second, it allows an immediate start in guiding patients through treatment possibilities and additional tests. Third, it provides novel perspectives on focal therapies. Patients diagnosed with PCa may potentially be treated in the same session and real-time detection of PCa from suspicious areas could make any prostate-sparing treatment easier and faster [29]. It offers the unique opportunity to improve the diagnostic pathway of PCa by linking together, with immediate feedbacks, urologists, radiologists, and pathologists. Finally, the fully digital nature of pathology and radiology images is particularly suited to the development of artificial intelligence methods for assisted diagnosis on both bpMRI and digital pathology images [30].

It is worth mentioning that this path does not necessarily include prostate MRI; in other words, real-time digital PCa diagnosis by FCM can obviously be carried out and achieved in patients scheduled for standard PBx.

Two studies tested the feasibility and diagnostic accuracy of FCM on prostate biopsy cores. Rocco et al. obtained digital images of biopsy cores in 54 patients. Perfect agreement between FCM and HE diagnosis was obtained for 95.1% of the 427 tested cores [17]. Similarly, Marenco et al. tested by FCM 182 MRI-targeted biopsy cores obtained in 57 biopsy-naïve patients. The median time for FCM processing and analysis was 5 min and the positive and negative predictive values were 85% and 95%, respectively [30]. Both the above-mentioned studies, however, focused on the “per core agreement” between FCM and HE. Indeed, not all biopsy cores taken were imaged using FCM. Conversely, by simultaneously taking images of three biopsy cores per single glass slide mounted, we were able to obtain a fast evaluation of 6 slides with up to 18 cores. As expected, we found that “per patient’s” agreement between FCM and HE diagnosis was even higher. Additionally, none of the previously mentioned studies reported agreement between FCM and HE staining in the grading of PCa. We found that GG was correctly classified in 90.8%, with a K coefficient of 0.80 (substantial agreement). Further studies including more patients and more than a single pathologist reviewing the slides are needed to assess the accuracy of FCM to evaluate Gleason Grading on a per patient level.

We acknowledge a few limitations of this study. The sample size was relatively small and there is a potential for inter-reader variability between the digital images to have occurred. Nevertheless, this pilot study was able to demonstrate the feasibility and efficacy of PCa diagnosis in one day as compared to the canonical diagnostic algorithm. Studies on a larger cohort will further determine the extent of the prognostic and oncological advantages of this promising single-day diagnostic pathway.

## 5. Conclusions

This pilot study indicated that “one-day” diagnosis of Pca using FCM was feasible and reliable. Though questions remain regarding the potential oncological advantages of such a pathway, there are no doubts about its advantages in optimizing time and resources, thus leading to patients’ perception of a high quality of care.

## Figures and Tables

**Table 1:** Clinical characteristics and biopsy results of patients included in the study. DRE: digitorectal examination, Q-Max: peak flow rate, P-vol: prostate volume, Bx GG: Biopsy Gleason Group.

							Digital FCM		Standard HE	
Pt.	Age	PSA	Q-Max	DRE	P-Vol	PI-RADS	Bx GG	Pos. Cores	Bx GG	Pos. Cores
1	50	3.71	32	Neg.	50	N/A	0	0	0	0
2	57	4.49	11	Neg	60	4	0	0	0	0
3	69	4.49	21	Susp	24	3	0	0	0	0
4	59	12	11	Susp	40	5	4	9	4	10
5	66	5.14	16	Neg	37	4	3	8	3	6
6	65	7.6	12	Susp	54	4	2	12	2	11
7	71	4.34	8	Susp	68	5	4	17	5	17
8	62	7.36	20	Neg	98	3	0	0	0	0
9	57	8.55	9	Neg	98	2	0	0	0	0
10	53	8.77	24	Susp	77	2	0	0	0	0
11	66	4.96	15	Susp	64	2	1	7	1	8
12	57	4.81	7	Susp	66	2	0	0	0	0

**Table 2:** Crosstabulation of per core Gleason Group (GG) assessment using digital FCM and standard of care (HE staining). Agreement and Cohen K statistics are reported for the detection of Any PCa (negative vs. GG 1-2-3-4-5), CsPCa (negative and GG 1 vs. GG 2-3-4-5), and individual grade groups (negative vs. GG 1 vs. GG 2 vs. GG 3, vs. GG 4, GG 5).

	Standard of Care (HE Staining)						
Vivascope	Negative	1	2	3	4	5	Total
Negative	128	2	0	2	1	0	133
1	2	9	1	0	1	0	13
2	0	0	2	3	0	0	5
3	0	0	1	7	2	0	10
4	0	0	0	0	20	2	22
5	0	0	0	0	0	2	2
Total	130	11	4	12	24	4	185
	Agreement	95% CI		Cohen K	95% CI		
Any PCa	0.962	(0.934, 0.990)		0.908	(0.841, 0.975)		
CsPCa	0.973	(0.949, 0.997)		0.922	(0.855, 0.990)		
Bx GG	0.908	(0.866, 0.950)		0.805	(0.722, 0.887)		

## Data Availability

The data that support the findings of this study are available on request from the corresponding author.

## Supplementary Materials

The following supporting information can be downloaded at: https://www.mdpi.com/article/10.3390/diagnostics12020277/s1, Supplementary methods: VivaScope 2500M-G4 Fluorescent confocal Microscope digital images staining protocol; Table S1: Patient Satisfaction Questionnaire results of the 12 patients included in the study; Supplementary Figure S1: Graphical abstract of the proposed One-Day Prostate cancer Diagnosis Path.
