# Supplementary material for: One-Day Prostate Cancer Diagnosis: Biparametric Magnetic Resonance Imaging and Digital Pathology by Fluorescence Confocal Microscopy
Source: Diagnostics (Basel). 2022 Jan 21;12(2):277. doi: 10.3390/diagnostics12020277 (PMC8871204; doi:10.3390/diagnostics12020277)

Supplementary Files

Supplementary methods:
VivaScope 2500M-G4 Fluorescent confocal Microscope digital images staining protocol

1) Saline Solution: 10 s
2) 70% Ethanol (it can be obtained by diluting the higher % ethanol, e.g. 95% in distill water): 20 s
3) Acridine Orange 1/50 Solution in distilled water: 20-30 s
4) 10% Acetic Acid (dilute the 20% acetic acid in saline solution): 20 s
5) Saline Solution: 10 s
6) Repeat 2-5
7) Partially dry the sample or the sample-sponge-complex on a tissue: it is better to use tissues that do not leave fibers on the sample (could generate artifacts)
8) Mount the sample: 100% Glycerol is applied on the sample surface to be imaged, or on the internal side of the microscope glass-slide (prior sample deposition).

Table S1. Patient Satisfaction Questionnaire results of the 12 patients included in the study.

The survey included 7 items about different aspects of care in patients with suspicion of PCa: 1. Time from suspicion to histological diagnosis; 2. Time from biopsy to histological diagnosis; 3. Workdays lost for PCa evaluation; 4. Reducing hospital visit during Covid-19 outbreak; 5. Prostate MRI; 6. Urological physical examination; 7. Blood tests.

Each item was scored with two subscales, ranging between 1 and 5, to assess perceived importance (not important (1) to extremely important (5)) and satisfaction with medical care (unsatisfied (1) to extremely satisfied (5))

	**Perceived importance (not important (1) to extremely important (5))**							**Satisfaction with medical care (unsatisfied (1) to extremely satisfied(5))**						
**ITEM**	**1**	**2**	**3**	**4**	**5**	**6**	**7**	**1**	**2**	**3**	**4**	**5**	**6**	**7**
**PZ1**	5	5	5	5	5	5	5	5	5	5	5	5	5	5
**PZ2**	5	5	5	5	5	5	5	5	5	5	5	5	5	5
**PZ3**	5	5	5	5	5	5	5	5	5	5	5	5	5	5
**PZ4**	2	2	3	5	5	3	3	3	2	4	2	2	3	3
**PZ5**	5	5	3	5	5	5	5	5	5	3	5	5	5	5
**PZ6**	5	5	5	5	5	5	5	5	5	5	5	5	5	5
**PZ7**	5	5	5	5	5	5	5	5	5	5	5	5	5	5
**PZ8**	5	5	3	5	5	5	5	5	5	3	5	5	5	5
**PZ9**	5	2	5	5	4	4	4	4	3	4	4	4	5	4
**PZ10**	5	5	5	5	5	5	5	5	5	5	5	5	5	5
**PZ11**	5	5	3	5	5	4	4	5	4	4	5	4	5	4
**PZ12**	5	5	3	5	5	4	4	5	3	4	5	4	5	4

Supplementary Figure S1.
Graphical abstract of the proposed One-Day Prostate cancer Diagnosis Path.
1. Biparametric MRI
2. Prostate Biopsy with target sampling of suspicious bpMRI lesions using an Electromagnetically Tracked MRI/US Fusion system
3. Real-time Digital pathology by fluorescent confocal Microscopy
4. Patients counselling about preliminary Biopsy results based on fluorescent confocal Microscopy.

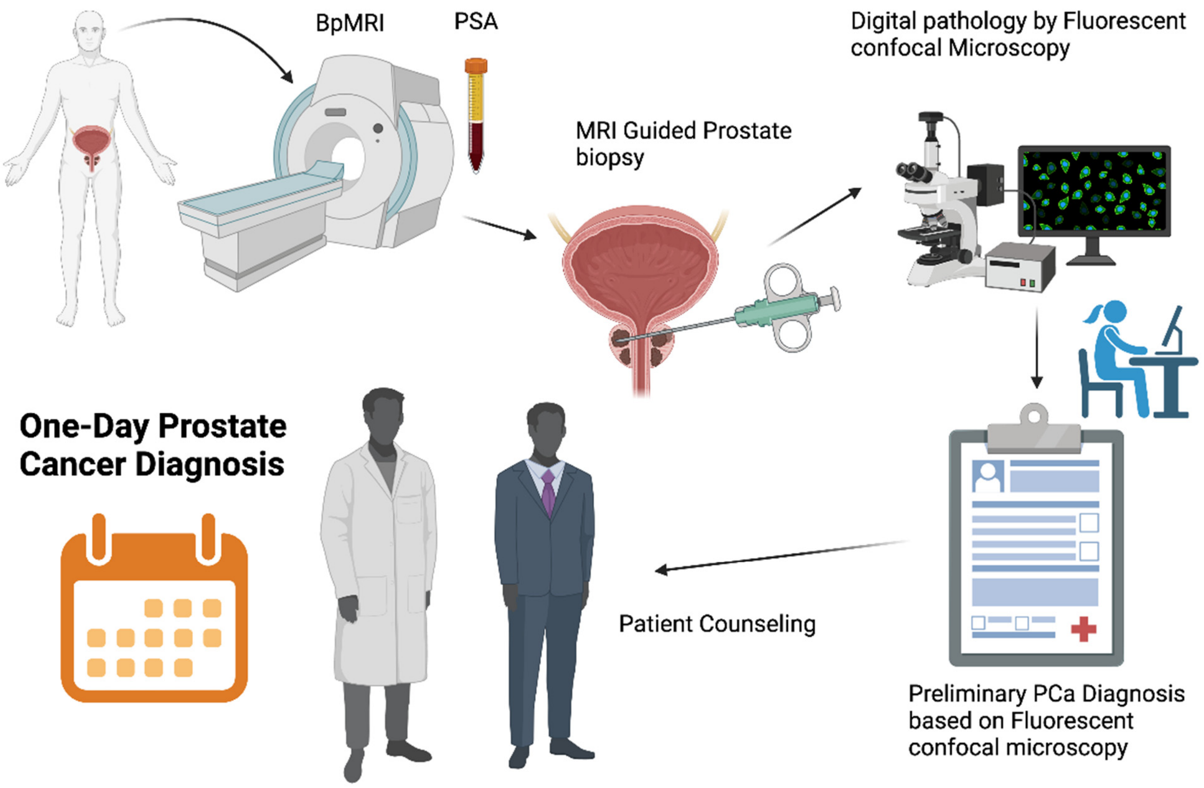

Supplement: Supplementary file 1 [file diagnostics-12-00277-s001.zip › diagnostics-1522984-supplementary.pdf]
